# Comments from the West

**Published:** 1893-08

**Authors:** 


### COMMENTS FROM THE WEST.

---

We have been pleased to see that several medical journals have commented upon efforts being made in Georgia to secure an Examining Board. It is needless to say that every one whose opinion is worth anything has been heartily in favor of the movement.

The following is from the Mississippi *Medical Monthly:*

The State that is now without a medical examining board is in a deplorable condition. Georgia is now reaping a harvest of these incompetents. It is estimated that of her twenty-seven hundred physicians, seven hundred are doctors rejected by boards of other States. No State in this Union can afford to be without a medical board, and we feel safe in predicting that no State will long be without one. It has been suggested that these rejected fellows will seek points of least resistance, and these points are States without examining boards. We are to be congratulated on our laws, regulating the practice of medicine, as well as an efficient board.

Out in Texas the situation seems to be very bad. We extend the right hand of sympathy and condolence to our brethren of the Lone Star State. A late number of the Texas *Sanitarian* says:

> Texas is in the same condition as Georgia, only more so. We have 4,000 physicians, and over 2,500 of this number would fail to pass any competent examining board in the land. About 1,600 of these incompetents have come to us within the last few years from other States having examining boards. Our contemporary (Mississippi *Medical Monthly*) would recognize many from Mississippi.

---

THE Pan-American Medical Congress meets in Chicago next month. Governor Northen has appointed the following delegates to represent Georgia: Robert Battey, C. P. Gordon, A. W. Calhoun, Dan Howell, W. P. Nicolson, K. P. Moore, J. C. Leahardy, J. W. Duncan, Eugene Foster, W. H. Dougherty, G. J. Grimes, I. H. Goss, A. H. Smith, Twiggs Miller, Reuben Nisbet, H. Holtzclaw, F. M. McIntosh, J. W. Bailey, P. R. Cortelyou, H. Perdue, —. Philpot, J. M. Kelley, W. C. Kendrick, J. B. S. Holmes, W. B. Tate, W. O'Daniel, W. S. Kendrick, S. Scott Todd, C. H. Hall, R. J. Nunn, J. H. Elliott, DeSaussure Ford, J. A. Dunwoody, —. Jordan, S. C. Benedict, N. P. Jelks, J. I. Darby, Lawrence Felder, J. G. Hopkins, A. M. Burt, J. H. Malone, —. Tennant, Frank M. Ridley, E. L. Bardwell, R. H. Taylor and N. G. Long.

